# Gut microbiota dysbiosis in COPD patients increases the level of queuine in the blood serum abnormally enhancing the viability of lung epithelial cells

**DOI:** 10.3389/fimmu.2026.1799075

**Published:** 2026-07-15

**Authors:** Yaozu Han, Zhaohui Mu, Lina Wang, Yunan Xu, Shiyang Chen, Chuanzhu Lv, Erdan Dong, Peng Yuan, Wei Han, Xinjuan Yu

**Affiliations:** 1 Clinical Research Center, Qingdao Hospital, University of Health and Rehabilitation Sciences (Qingdao Municipal Hospital), Qingdao, China; 2 Department of Respiratory and Critical Care Medicine, Qingdao Hospital, University of Health and Rehabilitation Sciences (Qingdao Municipal Hospital), Qingdao, China; 3 College of Medical Laboratory, Dalian Medical University, Dalian, China; 4 College of Clinical Medicine, Shandong Second Medical University, Weifang, China; 5 Emergency Medicine Center, Sichuan Provincial People’s Hospital, University of Electronic Science and Technology of China, Chengdu, China; 6 University of Health and Rehabilitation Sciences, Qingdao, China; 7 Department of General Practice, Qingdao Hospital, University of Health and Rehabilitation Sciences (Qingdao Municipal Hospital), Qingdao, China

**Keywords:** cell proliferation, chronic obstructive pulmonary disease (COPD), dysbiosis, gut microbiota, lung cancer, queuine (q), queuosine (Q)

## Abstract

**Introduction:**

To investigate the association between gut-airway microbiota dysbiosis, serum queuine levels, and early malignant transformation in patients with chronic obstructive pulmonary disease (COPD). We further explored whether the potential mechanistic role of queuine in enhancing lung epithelial cell viability under cigarette smoke exposure.

**Methods:**

Stable COPD patients were stratified into a high relative abundance of Proteobacteria group (CH) and a low relative abundance of Proteobacteria group (CL) using 16S rRNA gene sequencing of fecal samples. Airway microbiota profiles were analyzed in parallel to assess gut-lung axis coupling. Serum queuine concentrations were quantified using LC-MS/MS in healthy controls, COPD subgroups (CL and CH), and COPD patients complicated by lung cancer. Clinical symptoms (CAT, mMRC, SCSS) and spirometry (FEV_1_/FVC, FEV_1_, FEV_1_% predicted, FVC, FEF_25-75_%) were assessed. *In vitro* experiments were performed using cigarette smoke extract (CSE)-stimulated lung cancer epithelial A549 cells and bronchial epithelial BEAS-2B cells to determine the effects of queuine on cell viability. Chest CT imaging was analyzed to quantify pulmonary nodules as an indicator of *in vivo* epithelial proliferative activity.

**Results:**

The α-diversity of gut microbiota did not differ between CH and CL. In contrast, β-diversity showed separation (PERMANOVA P = 0.062), with CH characterized by Proteobacteria enrichment and relative depletion of Firmicutes, Bacteroidota, and Actinobacteriota. Airway communities showed concordant remodeling with shifts in taxa consistent with dysbiosis. Serum queuine concentrations increased stepwise from healthy controls to COPD, were higher in CH than CL, and were highest in COPD complicated by lung cancer. Despite comparable pulmonary function and symptom scores between CH and CL groups, the CH group exhibited a significantly higher number of pulmonary nodules on CT imaging, particularly ground-glass nodules. *In vitro*, queuine significantly enhanced the viability of CSE-stimulated A549 lung cancer cells but failed to rescue CSE-induced growth inhibition in BEAS-2B cells.

**Conclusion:**

COPD-associated gut microbiota dysbiosis, particularly enrichment of Proteobacteria, is closely associated with elevated systemic queuine levels. Excess queuine enhances cell viability of smoke-exposed lung cancer epithelial cells and is associated with increased pulmonary nodules *in vivo*. These findings identify queuine as a microbiota-derived metabolic mediator that may connect COPD-related dysbiosis to abnormal proliferation of lung epithelial cells.

## Graphical Abstract

**Figure:**
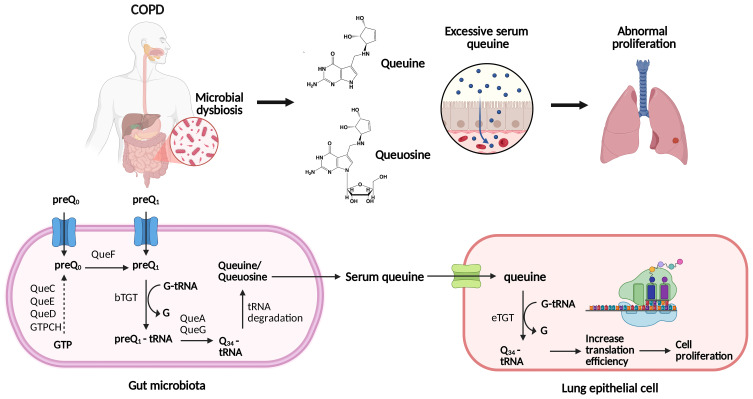
Gut microbiota-queuine-lung epithelial cell axis in COPD-associated abnormal epithelial proliferation.In COPD patients, gut microbiota dysbiosis may disturb queuine homeostasis by increasing queuine-producing bacteria, particularly Proteobacteria, and reducing queuine-salvaging bacteria such as Firmicutes, Bacteroidota, and Actinobacteriota. Elevated serum queuine may enter lung epithelial cells and participate in queuosine-modified tRNA formation, thereby enhancing translational efficiency and supporting abnormal epithelial growth under cigarette smoke- and inflammation-related stress. This study suggests a potential gut microbiota-queuine-lung epithelial cell axis linking COPD-related dysbiosis to increased pulmonary nodule burden and abnormal lung epithelial growth. COPD, chronic obstructive pulmonary disease; q, queuine; Q, queuosine; preQ0, 7-cyano-7-deazaguanine; preQ1, 7-aminomethyl-7-deazaguanine; GTP, guanosine triphosphate; tRNA, transfer RNA; Q-tRNA, queuosine-modified tRNA; TGT, tRNA-guanine transglycosylase; bTGT, bacterial tRNA-guanine transglycosylase; eTGT, eukaryotic tRNA-guanine transglycosylase; CSE, cigarette smoke extract; CH, COPD patients with relatively higher Proteobacteria abundance; CL, COPD patients with relatively lower Proteobacteria abundance.

## Introduction

Lung cancer remains one of the most consequential malignancies worldwide, with a persistently high mortality burden despite advances in screening and targeted or immune-based therapies (1, 2). Chronic obstructive pulmonary disease (COPD) is strongly linked to lung cancer occurrence and outcomes (2–4). Increasing evidence suggests that COPD does not merely co-exist as a shared consequence of smoking, but can contribute mechanistically to a lung milieu permissive for malignant transformation and progression (3, 5). Overall, approximately 40-70% of patients with lung cancer exhibit evidence of airflow limitation suggestive of COPD (3, 6), and COPD increases the risk of lung cancer by two- to seven fold, independent of smoking history (7, 8).

Queuine (q) and its tRNA modification product-queuosine (Q) provide a mechanistic link between the gut microbiome and host cellular programs. Queuosine is a hypermodified guanine-derived nucleoside found at the wobble position 34 of tRNAs decoding Asn, Asp, His, and Tyr codons (9, 10). Functionally, by incorporation Q into transfer RNA as a Q modification, Q accelerates the translation of NAU codons in mammalian cells, thereby enhancing translational efficiency (9, 11). Queuine availability is unusual among micronutrient. Some bacteria can synthesize the queuosine/queuine family *de novo* from GTP. This process involves sequential GTP-derived steps catalyzed by enzymes including FolE, QueD, QueE, QueC, and QueF to generate the precursor preQ1 (12, 13), which is then inserted into tRNA by bacterial TGT and further processed to Q-RNA (9). While others lack QueDECF proteins but still encode bTGT to salvage Q precursors preQ1 (14, 15). Unlike bacteria, which synthesize queuine *de novo* from GTP through a conserved enzymatic pathway, mammals lack this capacity and depend on dietary and microbial sources (9, 14). In eukaryotic cells, queuine is salvaged and incorporated into tRNA by the tRNA-guanine transglycosylase complex, making queuine availability directly dependent on intestinal microbial metabolism (14, 16). According to the Q synthesis, species of gut microbiome are composed of two microbial populations: Q-makers, mainly Proteobacteria, and Q-salvage, mainly Actinobacteriota, Bacteroidota and Firmicutes (17, 18). Maintaining an appropriate balance between these two populations may therefore be critical for stable queuine-dependent regulation of host physiology, whereas dysbiosis may shift net queuine supply.

With the increasing prevalence of COPD diagnoses, it has become evident that both the airway and gut microbiota of patients with COPD differ markedly from those of healthy individual. Bacterial community composition can display an increased representation of Proteobacteria with a relative reduction of Firmicutes and Bacteroidetes (19–21). However, despite the overall characteristic alterations in the gut microbiota of COPD patients, there is significant inter-individual microbiota heterogeneity, and studies have shown that COPD patients with different clinical phenotypes or disease stages may exhibit distinct dysbiosis patterns (22, 23). Here, in this study, we have differentiated COPD patients into two subgroups by gut microbiota composition, with subgroups exhibiting high relative abundance of Proteobacteria (CH) and low relative abundance of Proteobacteria (CL). We evaluated the association between microbial dysbiosis serum queuine levels, and early malignant transformation in patients with COPD, to explore the potential mechanistic role of queuine in promoting malignant transformation and progression of COPD.

## Materials and methods

### Participants

From March 2024 to December 2024, adult subjects with COPD were recruited in accordance with the criteria of the Global Chronic Obstructive Pulmonary Disease Initiative (GOLD). The diagnostic criteria are: the ratio of forced expiratory volume in one second to forced vital capacity (FEV_1_/FVC)<0.7. GOLD I: FEV_1_% >80%; GOLD II: FEV_1_% 50-80%; GOLD III: FEV_1_% 30-50%; GOLD IV: FEV_1_% <30%. The exclusion criteria are as follows: asthma, severe pneumonia, thoracic deformity, tuberculosis, pulmonary fibrosis, or other diseases that may affect pulmonary function test. The study was approved by the Ethics Committee of Qingdao Municipal Hospital. All patients signed the informed consent form before enrollment.

**Figure 1:**
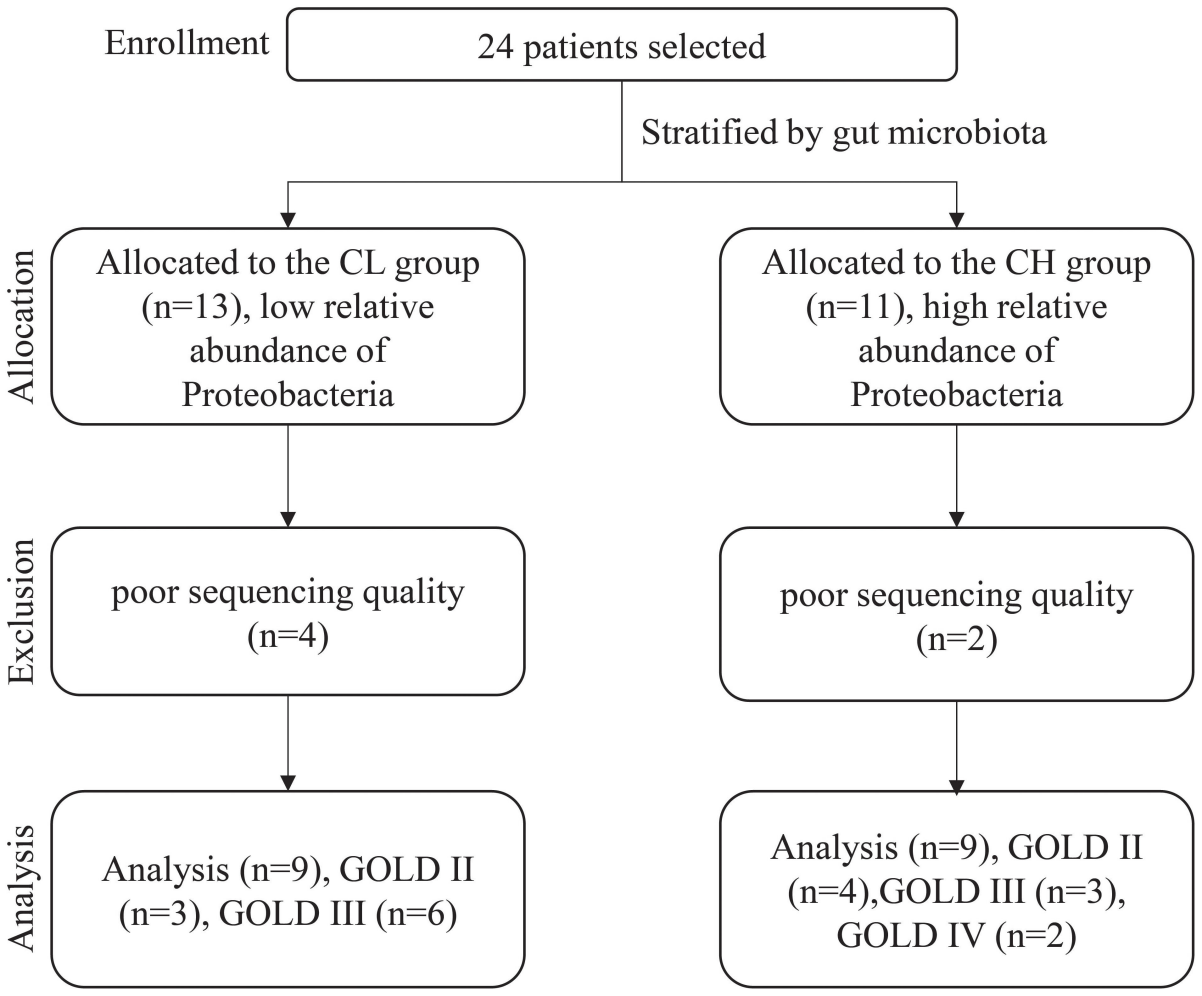
Study flow diagram. GOLD (Global Initiative for Chronic Obstructive Pulmonary Disease).

As shown in **Figure 1**, according to the data analysis of gut microbiota, all patients who met the inclusion criteria were divided into the CH group (COPD patients with high relative abundance of Proteobacteria in gut microbiota) and the CL group (COPD patients with low relative abundance of Proteobacteria in gut microbiota).

### Pulmonary function test

According to the guidelines of the American Thoracic Society/European Respiratory Society, all patients underwent bronchodilator pulmonary function tests (CHESTGRAPH HI-101, OMRON, Japan), including spirometry assessment. The test began 20 minutes after the subjects inhaled 400 μg of salbutamol. Evaluate the following variables: FVC, FEV_1_, FEV_1_/FVC, FEV_1_%, FEF_25-75_%.

### Chronic obstructive pulmonary disease assessment test questionnaire, modified medical research council dyspnea scale, semi-quantitative cough intensity score

Patients’ self-evaluation was conducted using the Chronic Obstructive Pulmonary Disease Assessment Test (CAT) questionnaire, the modified Medical Research Council dyspnea Scale (mMRC), and the Semi-quantitative Cough Intensity Score (SCSS). The CAT score ranges from 0 to 40. The lower the score, the fewer the symptoms. The mMRC classification ranges from 0 to 4 grades. The lower the score, the milder the symptoms of breathing difficulties. The SCSS score ranges from 0 to 5. The lower the score, the weaker the coughing ability.

### Quantification of serum queuine by LC-MS/MS

Queuine standard (Cat. No. IYT109287, Solarbio, China) was dissolved in the 50% methanol to prepare a 1 mM stock solution. Calibration standards were prepared by serial dilution of the stock solutions in 50% methanol to yield final concentrations of 5 nM, 10 nM, 20 nM, 50 nM, and 100 nM for making a standard curve. Serum samples were thawed at 4 °C, and 200 µL aliquots were transferred to microcentrifuge tubes. Proteins were precipitated by addition of 800 µL pre-cooled methanol/acetonitrile (1:1, v/v), followed by incubation at −20 °C for 2 h. Samples were then centrifuged to pellet precipitated proteins, and the supernatants were collected for LC-MS/MS analysis.

Chromatographic separation was performed on a Nexera X2 LC-30AD UHPLC system (Shimadzu) using a ACQUITY UPLC BEH C18 column (Waters, 1.7 µm, 2.1 mm × 100 mm). The autosampler was maintained at 4 °C and the column compartment at 40 °C. The flow rate was 300 µL/min and the injection volume was 1 µL. Mobile phase A consisted of water containing 0.1% (v/v) formic acid, and mobile phase B consisted of acetonitrile containing 0.1% (v/v) formic acid. The gradient program was: 0-3 min, 3-20% B (linear); 3-4 min, 20-90% B (linear); 4-6 min, 90% B (hold); 6-7 min, 90-3% B (linear); and 7-10 min, 3% B (re-equilibration). Mass spectrometric detection was carried out on an 5500 QTRAP instrument (AB SCIEX) equipped with an electrospray ionization (ESI) source operating in positive-ion mode. Quantification was performed using multiple reaction monitoring (MRM), monitoring the transition m/z 278.1/163.1 for queuine. Source parameters were set as follows: source temperature 550 °C; ion source gas 1 (GS1) 55; ion source gas 2 (GS2) 55; curtain gas (CUR) 35; and ion spray voltage (IS) 5500 V.

Data acquisition and processing were performed using Analyst software (v1.6.3). Chromatographic peak integration was based on retention time and peak-shape matching to the authentic standard. Peak area was used for quantification, and calibration curves were generated by linear regression of analyte peak area versus nominal concentration. Unknown sample concentrations were calculated by interpolation from the calibration model, with QC samples used to verify analytical performance across the batch.

### Cell culture and proliferation

The human normal bronchial epithelial cell line (BEAS-2B) and human NSCLC cell line (A549) were obtained from Procell Life Science & Technology Co., Ltd. (Wuhan, China). All cell lines were authenticated by short tandem repeat (STR) profiling and confirmed to be mycoplasma-free. BEAS-2B and A549 cells were cultured in medium supplemented with 10% fetal bovine serum (FBS; Cat. No. 164220, Pricella, Wuhan, China) and 1% penicillin-streptomycin (Cat. No. G4003, Servicebio, Wuhan, China). Cigarette smoke extract dissolved in the culture medium (CSE)was prepared by following previously reported procedures (24–26) and by combusting Kentucky Research Cigarettes (CODE 3R4F, Class A cigarettes, University of Kentucky, USA). Briefly, CSE was freshly prepared by bubbling the smoke from one research-grade cigarette into 25 mL of culture medium at a rate of one cigarette per 5 min. The resulting extract was passed through a 0.22 μm filter and defined as 100% (v/v) CSE.

Cell viability was assessed using the CCK-8 assay. Briefly, cells were seeded at a density of 1,800 cells per well in 96-well plates. After attachment, they were treated with fresh medium or the 5% or 10% of CSE for 24 h. After removing and washing with 1X PBS, they were treated with fresh medium or 1 nmol/L or 5 nmol/L queuine (dissolved in the culture medium). At 24 h post-treatment, 10 μL of CCK-8 reagent (Cat. No. 0005, Topscience, Shanghai, China) was added to each well and absorbance at 450 nm (OD_450_) was measured after 2 h of incubation at 37 °C.

### Image acquisition and feature extraction

All participants underwent standardized inspiration and breath-hold training provided by a professional technician prior to CT examination. During scanning, subjects were placed in a supine position with their hands above the head. Whole−lung CT scanning was performed from the lung apex to the base using a 64−slice spiral CT scanner during end−inspiratory breath−hold. The scanning parameters were as follows: tube voltage 120 kV, automatic tube current modulation, rotation time 0.6 s/rot, pitch 0.985, reconstruction slice thickness 1 mm or 1.25 mm, and continuous volume acquisition mode. All images were reconstructed using a standard algorithm.

The inspiratory thin−slice CT DICOM images of each subject were imported into the AI−based pulmonary nodule assisted diagnosis system for analysis. The following nodule characteristics were recorded from the software output: (1) maximum nodule diameter, (2) number of nodules, and (3) nodule density type (solid, part−solid, ground−glass).

### 16 rRNA gene sequencing and data analysis

Total cellular DNA was extracted from the collected fecal and sputum samples and determined based on the 16S region of bacteria. The highly variable regions V3 and V4 of prokaryotic 16S rDNA were amplified using universal primers (F: 5’ -ACTCCTACGGGGAGGCAGCA-3 ‘; Classify by R: 5’ GGACTACHVGGGGTWTCTAAT -3 ‘. The original data quality was filtered using Trimmomatic (27) (version 0.33), the primer sequence was identified and removed using Cutadapt (28) (version 1.9.1), and the two parts of the fragment were concatenated using USEARCH (29) (version 10). Finally, UCHIME (30) (version 8.1) was used to remove the chimeras to obtain high-quality sequences. In the qiime22020.6 software, the dada2 method was used for denoising to obtain the operational classification units (OTUs). The sequences were clustered using USEARCH (version 10.0) with a similarity of 97%, and the threshold was 0.005% of the total number of sequences filtered by the OTUs. Visualize the shared and unique features and numbers among samples using Venn diagrams (31).

Sequence data analysis was mainly conducted using QIIME2 2020.6 and the R package (v3.2.0). Taking SILVA [29] as the reference database, a simple Bayesian classifier (confidence level = 0.7) was used to annotate the feature sequences to obtain the corresponding species classification information. Then, the community composition of each sample was counted at the phylum and genus levels, and the species abundance table was generated using QIIME software. The community structure was analyzed using QIIME2 2020.6 and R software package (v3.2.0.1). The QIIME2 2020.6 (32) software was used to evaluate the Ace rich estimator and Shannon-Wiener diversity index in the Alpha diversity index at the OTU level. The species diversity matrix is constructed based on multiple algorithms such as binary jaccard, bray curtis, and (un) weighted unifrac (finite bacteria), and its eigenvalues and eigenvectors are sorted in the R language system based on principal coordinate analysis (PCoA). The abundance of significant differences between groups was analyzed by using LefSe (Line Discriminant Analysis Effect Size), and the taxonomic groups with significant differences were discovered. The LDA (Linear Discriminant Analysis) was used to estimate the degree of influence of the abundance of each component (species) on the differential effect. The Spearman correlation coefficient was used to analyze the correlation between the microbiota and metabolites and inflammatory factors. The scale of differential metabolites was not reduced, and two or more correlated variable factors were analyzed to measure the closeness of the correlation between two variable factors. Retain at least one set of correlation coefficients and correlation p-values that conform to: CC/> 0.8 and CCP< Set the data at 0.05 and then draw the heat map. by PICRUSt2 (Phylogenetic Investigation of Communities by Reconstruction of unobobserved States) 16S The reference sequence alignment of rrna with the microbial genome database was used to construct an evolutionary tree, which utilized the genotype and abundance information of known species to predict the genetic information of unknown species. The information of unknown species is combined with the KEGG pathway information of genes to predict the pathways of the entire community. The experimental data were statistically analyzed using GraphPad Prism 8.0. The data conforming to the normal distribution were expressed as mean ± standard deviation. Analysis of variance was used for the comparison between groups, and the Turkey method was used for the two-to-two comparison within the groups after the fact. For data with non-normal distribution, the mean ± standard deviation is expressed as the mean ± standard deviation.

### Statistical analysis

All statistical analyses were completed using SPSS 26.0 software. Categorical data were presented as counts (%), and chi-square test was used for comparison between groups. Continuous variables were expressed as mean ± standard deviation (SD) or mean ± standard error (SEM). Independent sample t-tests or the Mann-Whitney U test was used for comparison between two groups, and One-way analysis of variance (ANOVA) followed by Newman-Keuls test or the Kruskal-Wallis test followed by *post hoc* Dunn’s test was used for comparisons between three or more groups. P < 0.05 difference was considered statistically significant.

## Results

### Baseline characteristics

Baseline patients’ characteristics were detailed in **Table 1**. No significant difference was observed between CH group and CL group at the baseline data.

**Table 1:** Baseline characteristics of COPD patients.

	CH	CL	*χ*^2^/*z*/*t*-statistic	*P*
No. patients	9	9		
Male (%)	8 (0.84)	8 (0.84)	0	1
Age (years)	67.89 ± 5.84	66.78 ± 6.42	0.384	0.787
Height (cm)	173.44 ± 4.72	170.78 ± 7.67	0.888	0.400
Weight (kg)	74.56 ± 7.65	71.74 ± 11.27	0.636	0.427
BMI (kg/m^2^)	24.82 ± 2.70	24.71 ± 4.60	0.016	0.168
Smoking (%)	8 (0.89)	7 (0.78)	-0.603	0.231
FVC (L/S)	3.12 ± 0.62	2.70 ± 0.51	1.573	0.468
FEF_25-75_%	0.84 ± 0.46	0.76 ± 0.37	0.398	0.402
FEV_1_ (L/S)	1.68 ± 0.61	1.41 ± 0.44	1.088	0.339
FEV_1_% Predicated	0.53 ± 0.19	0.49 ± 0.15	0.561	0.382
FEV_1_/FVC	0.53 ± 0.13	0.51 ± 0.10	0.316	0.478
mMRC score *	2.00 ± 0.29	2.11 ± 0.11	-0.304	0.800
SCSS score	2.67 ± 0.50	2.78 ± 0.44	-0.500	0.332
CAT score	23.00 ± 3.60	23.56 ± 2.60	-0.375	0.138

Unmarked data are expressed as mean ± *SEM*; data marked with ‘*’ are expressed as mean ± *SD*; data followed by (%) are expressed as percentages.

FEV_1_ (Forced Expiratory Volume in 1 second); FVC (Forced Vital Capacity); FEF_25-75_% (Percentage of Predicted Forced Mid-Expiratory Flow); FEV_1_% (Percentage of Predicted Forced Expiratory Volume in 1 second); FEV_1_/FVC (Forced Expiratory Volume in 1 second/Forced Vital Capacity ratio); mMRC (Modified Medical Research Council Dyspnea Scale); SCSS (Semiquantitative Cough Strength Score); CAT (COPD Assessment Test).

### Structural characteristics of gut microbiota in COPD patients stratified by relative abundance of proteobacteria

**Figure 2:**
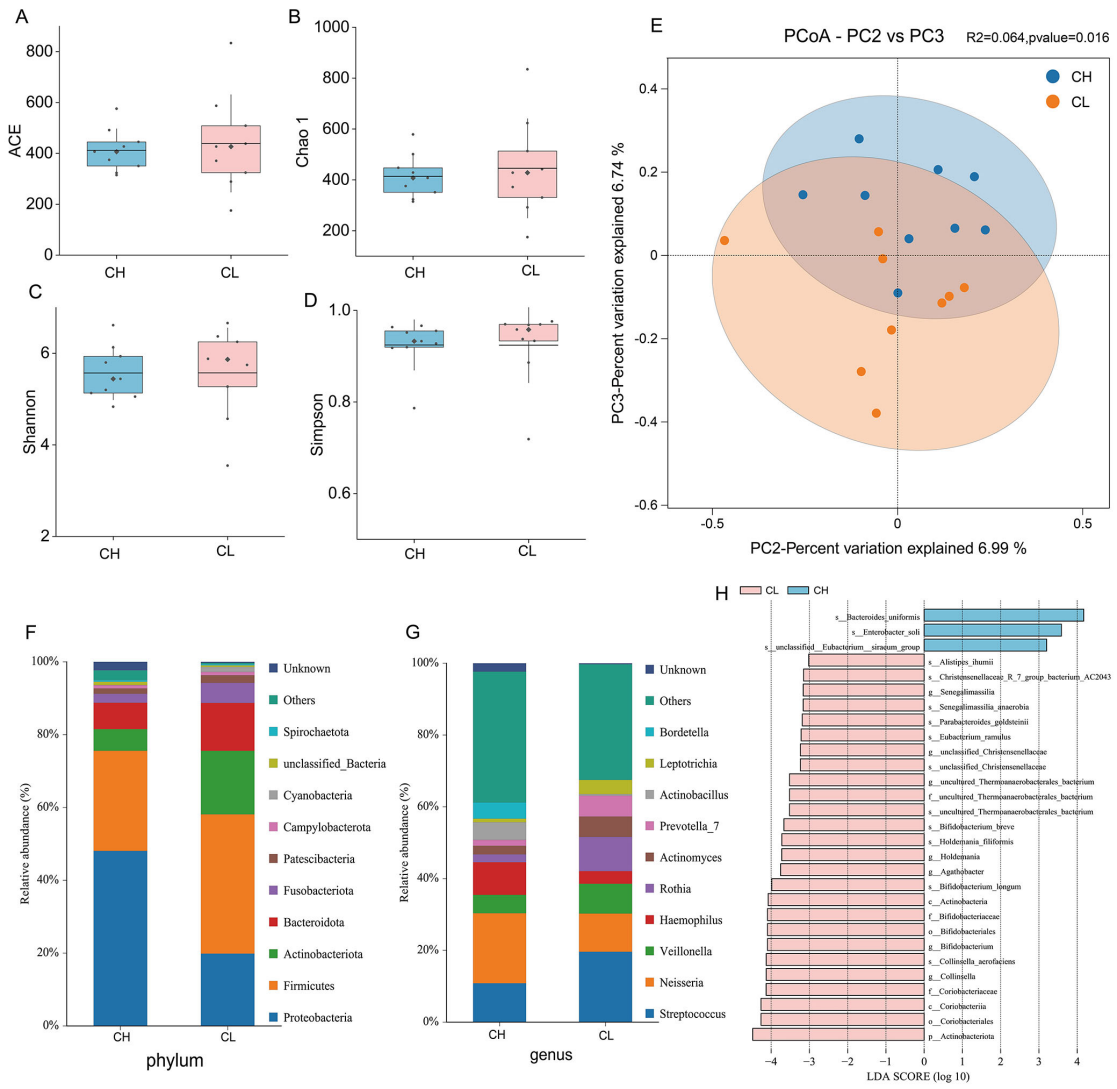
Gut microbiota composition and diversity analysis in COPD patients. **(A)** ACE index. **(B)** Chao 1 index. **(C)** Shannon index. **(D)**. Simpson index **(E)** Beta diversity analysis assessed by Principal Coordinates Analysis (PCoA), visualizing inter-sample dissimilarity based on microbial community structure. **(F, G)** Taxonomic distribution of Operational Taxonomic Units (OTUs): **(D)** Phylum-level and **(E)** genus-level OTU abundance, showing relative proportions of dominant bacterial groups. **(H)** Differentially abundant bacterial species identified by Linear Discriminant Analysis Effect Size (LEFSe) between pre- and post-treatment groups, highlighting taxa with significant shifts (LDA score >2.0).

Alpha diversity analysis of the gut microbiota showed no significant differences in Chao1, ACE, Shannon, or Simpson indices between the two COPD patient groups (all P>0.05) (**Figures 2A–D**); Beta diversity analysis indicated a significant separation of community composition between the two groups (PERMANOVA, P = 0.062, R²=0.016) (**Figure 2E**); At the phylum level, CH group exhibited decreased abundances of Firmicutes, Bacteroidota, Actinobacteriota, and Fusobacteriota except for Proteobacteria (**Figure 2F**). At the genus level, CH group showed reduced relative abundances of beneficial bacteria such as *Veillonella*, accompanied by increased abundances of harmful bacteria including *Haemophilus*, *Actinobacillus*, and *Neisseria* (**Figure 2G**). LEfSe analysis further confirmed that the relative abundances of potential beneficial bacteria, including *Parabacteroides goldsteinii*, *Bifidobacterium breve*, *Bifidobacterium longum*, *Bifidobacteriaceae*, *Bifidobacteriales*, and *Bifidobacterium*, were elevated in the CL group compared to the CH group (**Figure 2H**). Under conditions of high gut Proteobacteria abundance, the gut microbiota structure showed a decrease in the relative abundance of potentially beneficial bacteria and an increase in the relative abundance of potentially harmful bacteria, failing to shift toward homeostasis improvement and instead exhibiting exacerbated dysbiosis.

### Structural characteristics of airway microbiota associated with gut microbiota phenotype via gut-lung axis

**Figure 3:**
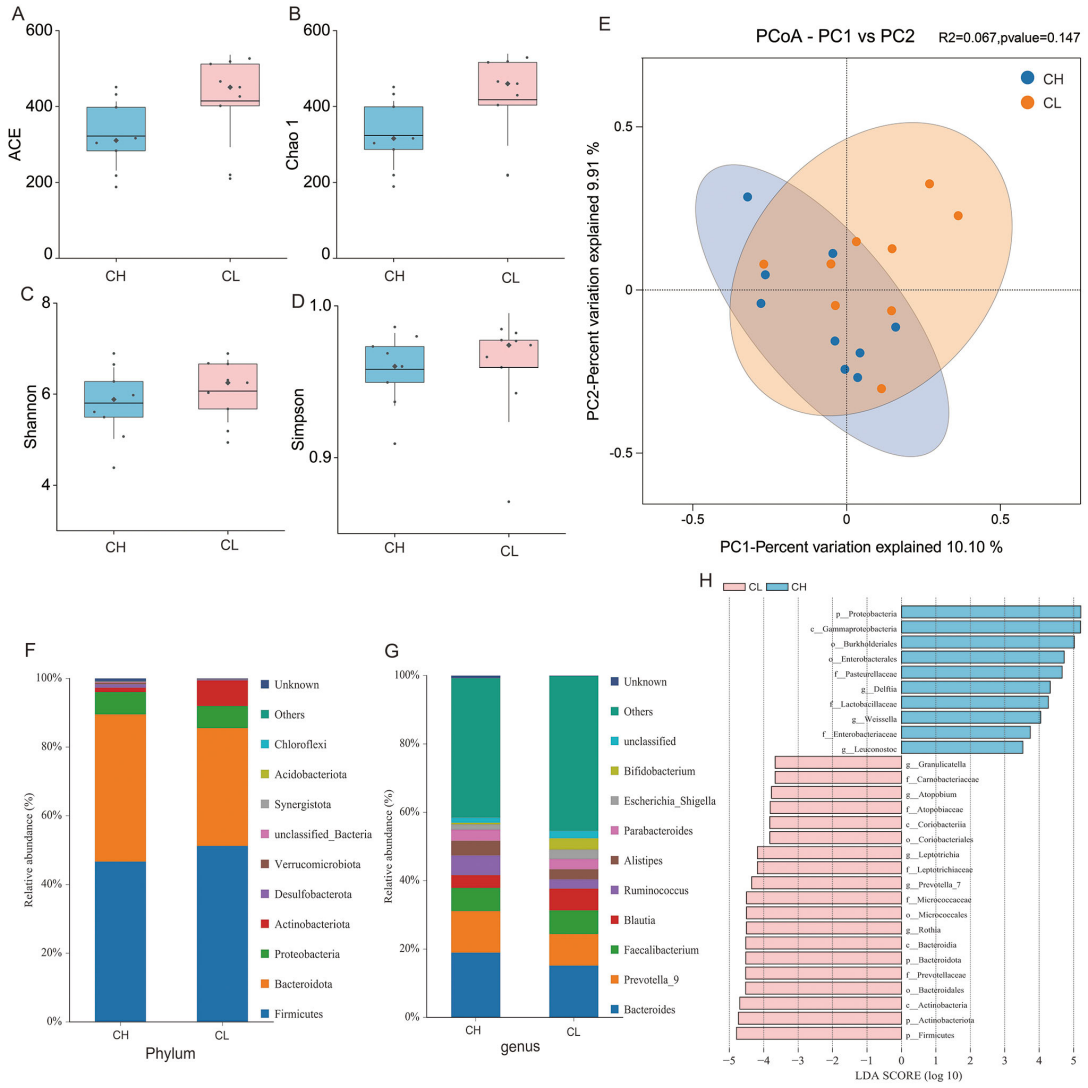
Airway microbiota composition and diversity analysis in COPD patients. **(A)** ACE index. **(B)** Chao 1 index. **(C)** Shannon index. **(D)**. Simpson index **(E)** Beta diversity analysis assessed by Principal Coordinates Analysis (PCoA), visualizing inter-sample dissimilarity based on microbial community structure. **(F, G)** Taxonomic distribution of Operational Taxonomic Units (OTUs): **(D)** Phylum-level and **(E)** genus-level OTU abundance, showing relative proportions of dominant bacterial groups. **(H)** Differentially abundant bacterial species identified by Linear Discriminant Analysis Effect Size (LEFSe) between pre- and post-treatment groups, highlighting taxa with significant shifts (LDA score >3.5). Data represent mean ± *SEM*.

Alpha diversity analysis of the airway microbiota revealed no significant changes in the Shannon or Simpson indices under conditions of high abundance of Proteobacteria in gut (all *P*>0.05). Meanwhile, the ACE and Chao 1 indices showed a downward trend in the CH group compared to the CL group, although the differences did not reach statistical significance (P = 0.086 and P = 0.08, respectively) (**Figures 3A–D**); Beta diversity analysis indicated a trend of separation in community composition between the two groups (PERMANOVA, *P* = 0.147, R²=0.067) (**Figure 3E**); At the phylum level, the relative abundances of Acidobacteriota and Firmicutes decreased in the CH group, while those of Bacteroidota increased (**Figure 3F**); at the genus level, the relative abundances of beneficial bacteria such as *Blautia* and *Bifidobacterium* increased in the CL group (**Figure 3G**); LEfSe analysis further confirmed that in the CL group, the relative abundances of potential beneficial bacteria such as *Bacteroidota*, *Bacteroidia*, *Bacteroidales*, *Prevotellacea*, and *Prevotella_7* increased, while in the CH group, those of potential harmful bacteria such as *Gammaproteobacteria* and *Enterobacteriaceae* increased (**Figure 3H**). These results suggest that under conditions of high gut Proteobacteria abundance, the airway microbiota composition is altered, with a decrease in beneficial bacteria and an increase in harmful bacteria, thereby reducing airway microbiota diversity and disrupting its ecological balance.

### Effects of gut microbiota phenotype in COPD patients on the symptoms and lung function

**Figure 4:**
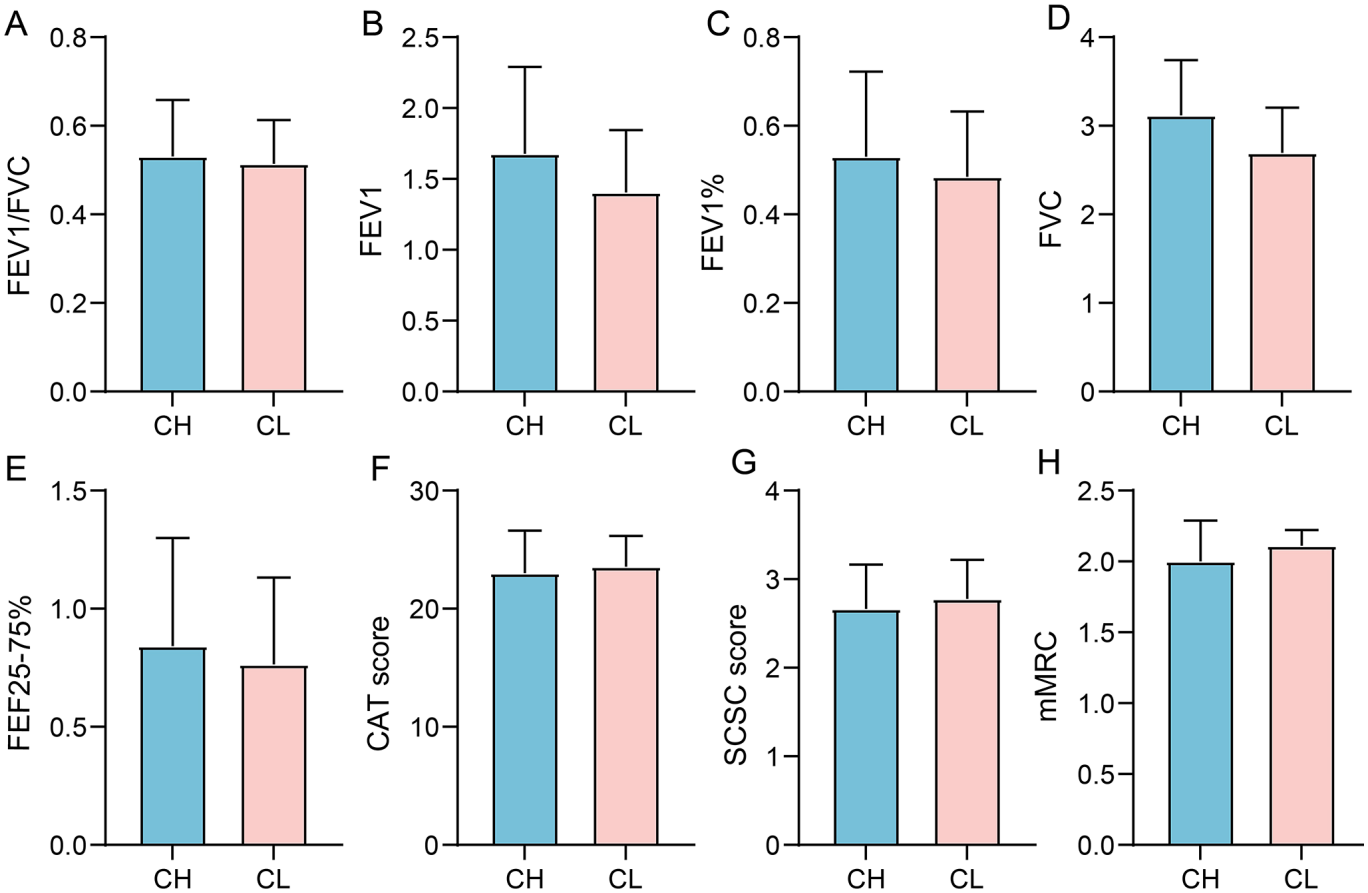
Effects of the gut abundance of Proteobacteria in COPD patients on the clinical symptoms and pulmonary function. **(A–E)** Comparison of pulmonary function parameters: **(A)** FEV_1_/FVC, Forced Expiratory Volume in 1 second/Forced Vital Capacity ratio; **(B)** FEV_1_, Forced Expiratory Volume in 1 second; **(C)** FEV_1_%, Percentage of Predicted Forced Expiratory Volume in 1 second; **(D)** FVC, Forced Vital Capacity; **(E)** FEF_25-75_%, Percentage of Predicted Forced Mid-Expiratory Flow. **(F–H)** Comparison of symptom scores between the two groups: **(F)** CAT, COPD Assessment Test; **(G)** SCSS, Semiquantitative Cough Strength Score; **(H)** mMRC, Modified Medical Research Council Dyspnea Scale.

To evaluate the effect of gut microbiota phenotype in COPD patients on the lung function in COPD patients, we assessed clinical symptoms using pulmonary function indices and the mMRC, SCSS, and CAT scales. There were no significant differences in pulmonary function indices (FEV_1_/FVC, FEV_1_, FEV_1_%, FVC, FEF_25-75_%) between the CL and CH group (all *P*>0.05) (**Figures 4A–E**), and no significant differences were observed in mMRC, SCSS, or CAT scores (all *P*>0.05) (**Figures 4F–H**).

### Serum queuine levels progressively elevated from healthy controls to COPD complicated with lung cancer

**Figure 5:**
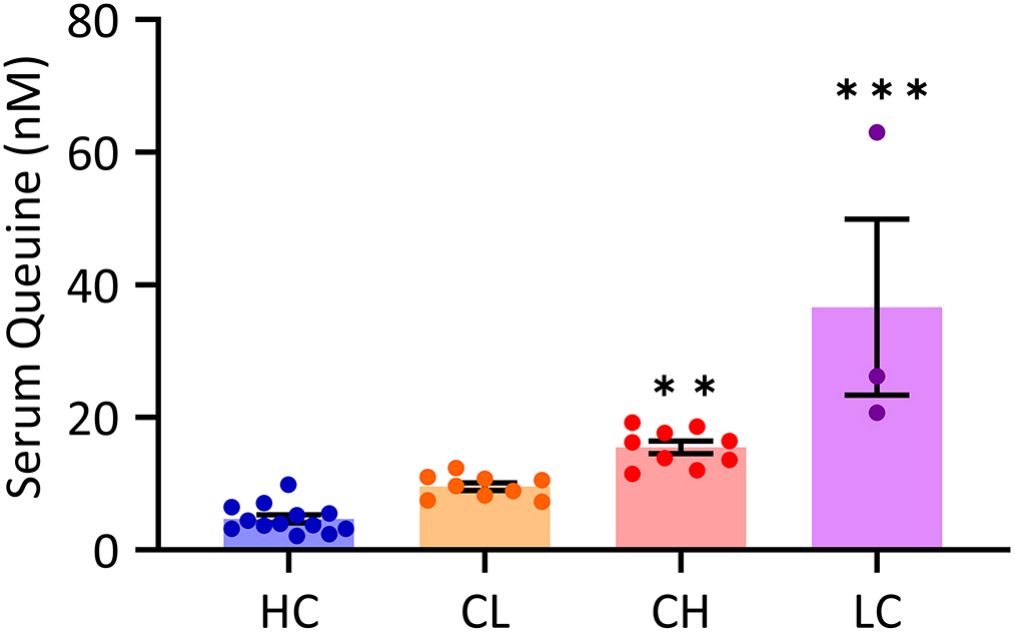
Serum queuine levels progressively elevated from healthy controls to COPD complicated with lung cancer. Serum queuine concentrations were measured by LC-MS/MS method in HC, CL, CH, and LC groups. Each point represents an individual participant and the data are presented as mean ± *SEM*. *vs* HC, *P < 0.05,**P < 0.01, ***P < 0.001.

Microbial dysbiosis in COPD alters the balance between Q-makers and Q-salvages bacterial population, which shows a significant increase in relative abundance of Q-makers bacteria and an decrease in relative abundance of Q-salvages (17, 18). To investigate whether gut microbiota alterations were associated with queuine availability, we collected blood samples and quantified serum queuine concentrations from healthy controls (HC), COPD patients complicated by lung cancer (LC), as well as COPD patients further stratified by gut microbiota phenotypes (CL and CH). Quantification of serum queuine level shows healthy individuals exhibited uniformly low baseline levels of serum queuine, with minimal inter-individual variability (**Figure 5**). In contrast, COPD patients displayed an elevation in serum queuine compared with healthy controls. This increase was not uniform across subgroups: patients classified within the CH group exhibited significantly higher serum queuine concentrations than those in HC, while patients in the CL group seemed higher but no statistical significance. And, compared to CL group, serum queuine levels were higher in the CH group (**Figure 5**). This stepwise increase indicates a close association between disease severity, microbial communities and systemic queuine accumulation. And, COPD-related dysbiosis of the gut microbiome contributes to the altered queuine level in the host circulation.

To explore whether queuine dysregulation is linked to COPD malignant transformation, we analyzed serum samples from COPD patients complicated by lung cancer. Strikingly, serum queuine levels were further elevated in the lang cancer patients compared with all other groups, including CH patients (**Figure 5**). This consistently increase may suggest a mechanistic link between elevated serum queuine and the transition from COPD to early-stage lung cancer and sustained queuine excess may promote lung cancer initiation and growth.

### Queuine enhances the viability of CSE-induced lung cancer epithelial A549 cells

**Figure 6:**
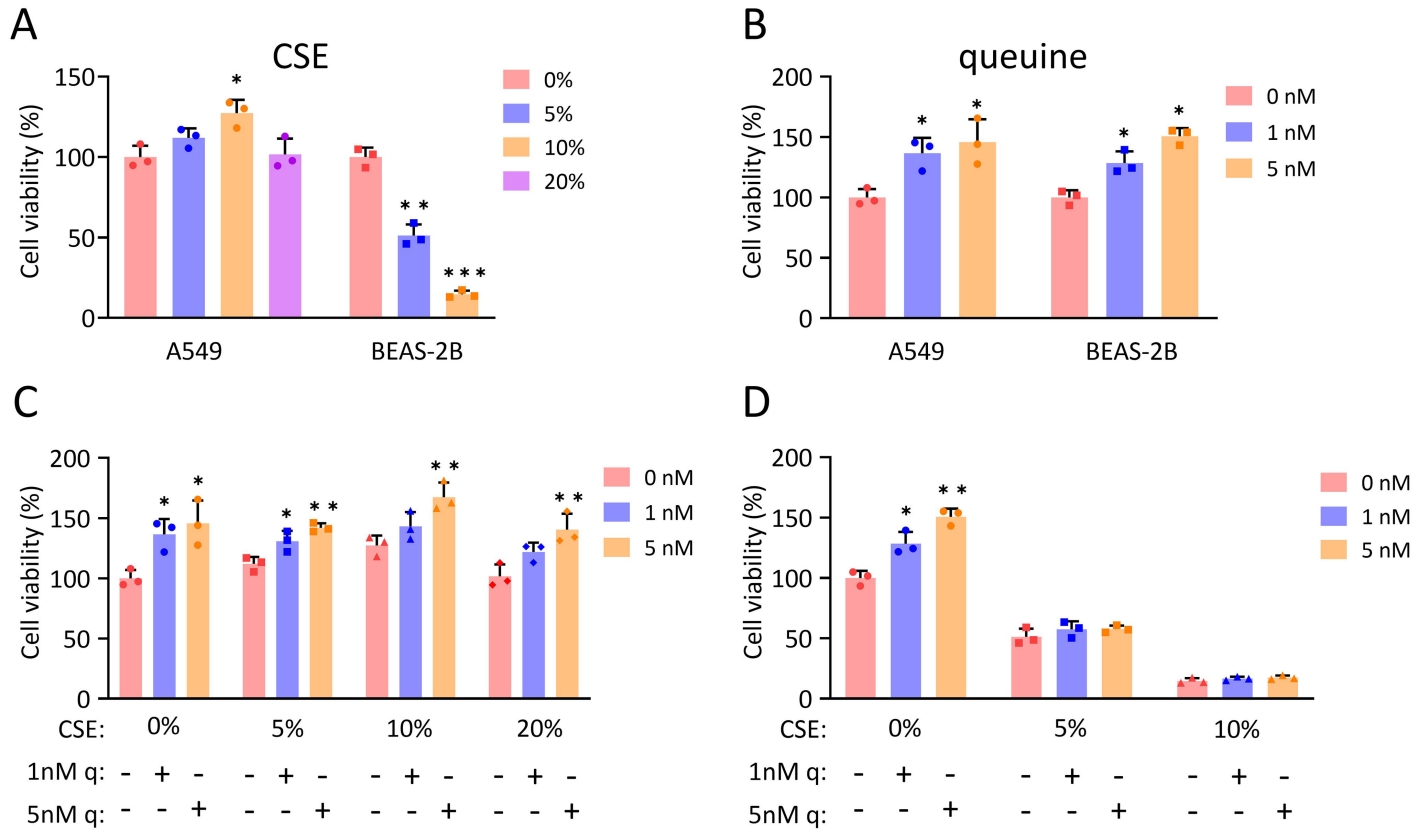
Queuine significantly promotes the viability of A549 cells under CSE stimulation. **(A)** Cell viability of A549 and BEAS-2B cells after exposure to increasing concentrations (0%, 5%, 10%, or 20%) of CSE for 24 h. CCK8 assay was used to evaluate cell viability. **(B)** Cell viability of A549 and BEAS-2B cells after treatment with concentrations of 0, 1, or 5 nM queuine for 24 h. **(C, D)** Cell viability of A549 and BEAS-2B cells after pre-exposed to CSE at varying concentrations (0%, 5%, 10%, 20%) for 24 h, and then treated with concentrations of 0, 1, or 5 nM queuine for incubation 24 h. Each point represents an individual participant and the data are presented as mean ± *SEM*. Significance: *P < 0.05, **P < 0.01, ***P < 0.001.

To determine whether queuine enhances the growth of the lung cancer cells under cigarette smoke exposure, we first established a CSE-stimulated cell model by treating lung cancer epithelial A549 cells and lung epithelial BEAS-2B cells. Cells were treated with increasing concentrations of CSE (0%, 5%, 10%, and 20%) for 24 h. The CCK-8 assay demonstrated that, BEAS-2B cells exhibited a marked loss of viability following CSE exposure. Specifically, 5% CSE significantly inhibited viability of BEAS-2B cells, and this suppressive effect intensified with higher CSE concentrations. Rather than uniformly suppressing growth, 10% CSE significantly enhanced A549 cell viability compared with untreated controls (**Figure 6A**). Even at 20% CSE, no significant growth inhibition was observed in A549 cells, suggesting smoke selectively promotes apoptosis and growth inhibition in BEAS-2B cells while conferring a proliferative advantage to A549 cells. We next examined the effect of queuine on the viability of CSE-stimulated or untreated A549 and BEAS-2B cells. The results showed that, after supplementation for 24 h, both 1 nM and 5 nM queuine significantly increased the viability of A549 and BEAS-2B cells (**Figure 6B**). Furthermore, after treatment with CSE for 24 h, A549 and BEAS-2B cells were supplemented with 1 nM or 5 nM queuine and continued to culture for an additional 24 h. As shown in **Figure 6C**, compared to CSE-only groups, queuine consistently promoted the viability of A549 cells across CSE-stimulation conditions. The effect was particularly robust for 5 nM queuine, which significantly enhanced cell growth even under 20% CSE stimulation, effectively overriding the adverse effects of high-dose cigarette smoke exposure. By contrast, the viability of BEAS-2B cells was markedly inhibited by CSE and subsequent queuine treatment failed to rescue cell viability (**Figure 6D**). Although queuine alone increased BEAS-2B cells growth under baseline conditions, its inability to reverse CSE-mediated suppression. These findings demonstrate that queuine enhances viability of smoke-exposed lung cancer epithelial cells, particularly at higher concentrations.

### Increased pulmonary nodules in COPD patients with high serum queuine level

**Figure 7:**
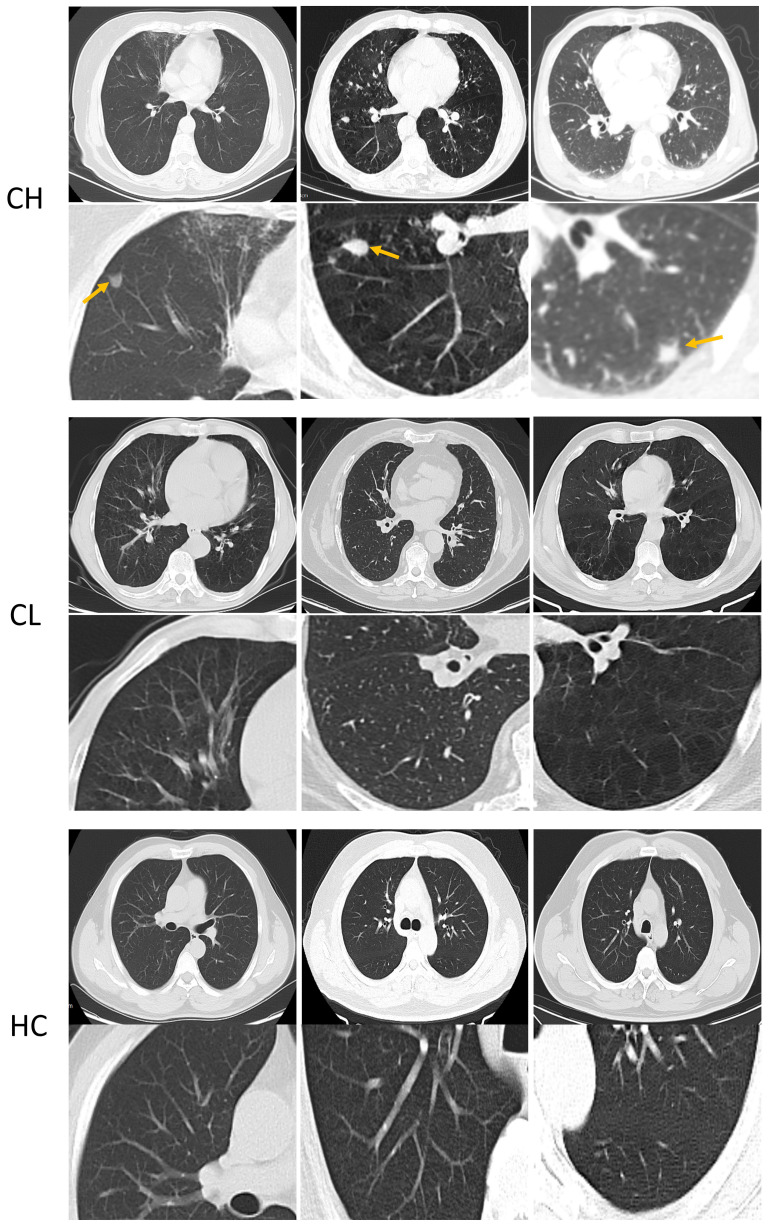
Representative chest CT images between the CH and CL groups. Representative chest CT images are shown for the CH group (top) and CL group (bottom), with three examples per group (left to right). For each example, the upper row shows the original axial CT image, and the lower row shows a magnified view of the region of nodules. Yellow arrows indicate a marked pulmonary nodule in each case of the CH group.

To evaluate *in vivo* lung cell proliferation in COPD patients, we quantified pulmonary nodules on chest CT as an imaging-based surrogate of ongoing epithelial outgrowth. As shown in **Table 2**, we compared total number of nodule, mean nodule diameter, and radiological feature subtypes, including ground-glass nodules, partially solid nodules, and solid nodules. Across the cohort, the total number of pulmonary nodules and the number of ground-glass nodules were both significantly higher in the CH group compared with the CH group (P = 0.002 and P = 0.015, respectively). While, the mean nodule diameter and the numbers of partially solid and solid nodules did not reach statistical significance (all P > 0.05). Representative CT images are shown in **Figure 7**, which visually illustrates the differences in pulmonary nodule manifestations between the CH and CL groups, suggesting that the increase in pulmonary nodules may be associated elevated serum queuine.

**Table 2:** Data on pulmonary nodules in COPD patients.

	CH	CL	*χ*^2^/*z*/*t*-statistic	*P*
Total nodules *	23.00 ± 6.45	5.67 ± 0.42	-2.903	0.002
Nodule diameter(mm)	8.47 ± 1.71	3.52 ± 0.29	2.859	0.082
Ground-glass nodules *	7.83 ± 5.87	0.33 ± 0.21	-2.539	0.015
Partially solid nodules *	1.50 ± 0.67	0.33 ± 0.21	-1.396	0.163
Solid nodules	13.67 ± 1.99	5.00 ± 0.45	4.240	0.102

Unmarked data are expressed as mean ± SEM; data marked with ‘*’ are expressed as mean ± SD.

## Discussion

Here, we investigated whether COPD-associated gut dysbiosis reshapes host queuine availability and thereby contributes to an abnormal proliferation in lung epithelial cells that may facilitate COPD-to-lung cancer transformation. Our data suggest that this is the case. Specifically, we found a microbiome-based stratification of COPD by Proteobacteria abundance, defining a high and low abundance of Proteobacteria subgroup. Although COPD is often treated as a single clinical entity, accumulating evidence indicates marked inter-individual heterogeneity in microbial configurations (22, 23). This stratification was accompanied by a reciprocal shift in other major phyla, most notably reduced Bacteroidetes and Firmicutes in the CH subgroup. However, lung function and symptom scores did not differ significantly between CH and CL subgroup. We do not have an obvious explanation, but it is consistent with a growing view that lung function reflect cumulative and often slowly evolving structural determinants that may not track with gut microbial composition measured cross-sectionally (33).

A central finding of this study was the stepwise elevation of serum queuine across disease states, from healthy controls to COPD and further to COPD complicated by lung cancer, with an additional increase in the CH group compared with CL group. Cigarette smoke may contribute to elevated serum queuine levels through several indirect mechanisms. First, cigarette smoke exposure can disturb both airway and gut microbial communities (20, 34). Enrichment of Proteobacteria may increase the proportion of bacterial taxa capable of *de novo* queuine/queuosine biosynthesis, whereas depletion of Firmicutes and Bacteroidota may reduce queuine-salvaging capacity. This imbalance may increase the net systemic availability of microbiota-derived queuine. Second, cigarette smoke and COPD-related inflammation may impair epithelial barrier integrity and alter microbial metabolite flux along the gut-lung axis (35, 36). This may facilitate the absorption or translocation of microbiota-derived metabolites, including queuine, into host circulation. Therefore, cigarette smoke is unlikely to directly activate queuine chemically. Instead, it may create a dysbiotic and barrier-disrupted microenvironment that favors systemic queuine accumulation.

Elevated queuine may also participate in COPD-related epithelial remodeling through tRNA modification-dependent translational regulation. Mammalian cells cannot synthesize queuine *de novo* and depend on dietary and microbial sources for queuosine-tRNA modification (9, 16). This modification regulates codon decoding, translational efficiency, protein homeostasis, mitochondrial stress responses, and cellular adaptation to oxidative stress (9, 16, 37). Recent studies have shown that mammalian queuosine-tRNA modification can enhance translational activity and cell viability (11, 38). In COPD, chronic cigarette smoke exposure induces persistent oxidative stress, epithelial injury, inflammation, and repeated repair responses. Under these conditions, increased queuine availability may support translational adaptation and survival of stress-tolerant epithelial cell populations. This mechanism may help explain why queuine enhanced the viability of CSE-exposed A549 cells in our study, whereas it did not rescue CSE-induced injury in BEAS-2B cells. However, direct evidence that queuine initiates COPD is currently lacking. Thus, we interpret queuine as a potential microbiota-derived metabolic mediator that may amplify COPD-related epithelial remodeling and abnormal epithelial growth, rather than as an independent cause of COPD.

Also, Queuosine modification may influence cancer-related biological processes, including cell-cycle control, DNA repair, PI3K/Akt signaling, p53 regulation, proteostasis, and cell proliferation (10, 39). Notably, Díaz-Rullo et al. reported that depletion of queuine/queuosine modification increased Akt activation and affected p53-related regulation in human cancer cells, supporting a potential link between queuine status and tumor-associated proliferative signaling (10). In addition, recent work showed that mammalian queuosine tRNA modification can enhance translation and cell proliferation, whereas the bacterial precursor preQ1 can compete with queuine for tRNA modification and suppress mammalian cell proliferation and tumor growth in a mouse cancer model (11, 38). These findings indicate that queuine-related metabolites may exert bidirectional and context-dependent effects on tumor cell growth, depending on intracellular uptake, tRNA modification status, and the balance between queuine and related microbial intermediates.

The altered level of queuine during lung epithelial malignant transformation provides additional evidence that queuine may participate in early epithelial carcinogenic events rather than only in established tumor proliferation. This interpretation is supported by recent evidence that queuine/queuosine availability depends on bacterial synthesis, dietary input, and host uptake pathways, including the high-specificity transporter SLC35F2, which has been identified as a transporter for queuine and queuosine (40). Moreover, QTRT1, the catalytic component of the queuine tRNA-ribosyltransferase complex, has been implicated in tumor biology and epithelial cell proliferation; QTRT1-related queuosine modification was reported to regulate genes involved in proliferation, tight junction formation, and migration, while altered QTRT1 expression has also been associated with lung adenocarcinoma prognosis (39, 41, 42). Therefore, compared with previous studies mainly focusing on tumor cell proliferation, our findings highlight a more specific role of queuine in lung epithelial malignant transformation. Queuine may represent a metabolic node connecting respiratory microbial dysbiosis, epithelial translational remodeling, barrier dysfunction, oxidative stress, and malignant transformation. Nevertheless, because the current study is primarily observational, further functional experiments are required to determine whether queuine directly regulates lung epithelial transformation through QTRT1-mediated tRNA modification, Akt/p53 signaling, or microbiome-dependent metabolic pathways.

Although this study elucidates the association between Proteobacteria enrichment, serum queuine elevation, and abnormal proliferation of lung epithelial cells, limitations should be considered. Firstly, the sample size was relatively small, with only 9 participants included in each COPD subgroup. Consequently, certain microbiome-related analyses, particularly β-diversity comparisons, demonstrated only marginal statistical significance. Therefore, the present findings should be interpreted cautiously and regarded as exploratory rather than definitive evidence of causality. Proteobacteria enrichment and increased serum queuine may also be co-driven by unmeasured confounding factors. Future large-scale, multicenter cohort studies with well-matched clinical variables are needed to validate the robustness and reproducibility of these findings. Second, this study did not include animal experiments; thus, the causal contribution of specific microbial communities or microbiota-derived metabolites to lung epithelial malignant transformation could not be directly confirmed *in vivo*. Future studies should establish appropriate animal models, such as microbiota transplantation models, carcinogen-induced lung tumorigenesis models, or germ-free/antibiotic-treated mouse models, to determine whether microbiome modulation that reduces Proteobacteria abundance can decrease serum queuine levels and attenuate nodule development or epithelial malignant transformation over time. Third, although serum queuine was quantified by LC-MS/MS, we did not directly assess microbial queuine biosynthesis or salvage genes and their transcriptional activity, nor did we measure host tRNA queuosine modification levels in blood or airway epithelial cells. These measurements would help strengthen the mechanistic chain from microbial community alterations to circulating queuine changes and subsequent functional translational regulation. In addition, further molecular studies are required to clarify whether candidate microbiota-derived metabolites regulate epithelial proliferation, oxidative stress, inflammatory signaling, DNA damage responses, epithelial-mesenchymal transition, immune microenvironment remodeling, or tRNA modification-related translational control. Finally, future work should focus on targeted intervention of key microbiota-derived metabolites and evaluate their clinical translational potential as early diagnostic biomarkers, risk-stratification indicators, or preventive and therapeutic targets for lung epithelial malignant transformation. These additional studies will help move the current observational findings toward mechanistic clarification and clinical application.

## Data Availability

Source data for microbiome sequencing have been deposited to the NCBI BioProject database (accession: PRJNA1420362), respectively.
